# Fellow-Workers and Their Doings

**Published:** 1914-03-07

**Authors:** 


### ROUND THE WORLD.

### Fellow-Workers and their Doings.

[The Editor Invites Early Information and Contributions Marked "Fellow-Workers."]

Mr. C. A. Joll, F.R.C.S., who has just been elected assistant surgeon to the Royal Free Hospital (School of Medicine for Women), had a brilliant academic career as a student at Bristol, University College, and the London Hospital. At the M.B., B.S. Lond. Examination in 1911 he took the gold medal with honours in four subjects. Mr. Joll took his further degree of B.Sc. in 1905, following this up with the Mastership in Surgery and the Fellowship of the Royal College of Surgeons. In addition to these distinctions he holds the medical and surgical degrees of Bristol University and the Royal College of Surgeons' licence to practise dental surgery. Mr. Joll has served his apprenticeship at the Royal Free Hospital in the capacity of resident medical officer.

---

During the last two years Dr. Henry Head, M.D., F.R.S., the well-known neurologist, has been devoting a good deal of time at the London Hospital and elsewhere to the problems of parasyphilitic nervous diseases. The results of his investigations when published will throw a good deal of light on the problems of locomotor ataxy and general paralysis of the insane. An important practical point that has been revealed in the course of Dr. Head's work is that great improvement, indicated both clinically and by laboratory tests, can be secured in such conditions by intravenous injections of salvarsan, which, however, to be efficacious must be repeated. Another point these researches are helping to make clear is the distinction between simple and specific spinal meningitis.

---

Dr. William Permewan, M.D., F.R.C.S., has added to his previous successes as an eminent provincial laryngologist by being appointed lecturer in his special subject at Liverpool University. Dr. Permewan is an old University College Hospital student, and includes among his present appointments those of surgeon to the Royal Southern Hospital, Liverpool, and throat and ear surgeon to the Southport Infirmary.

---

Mr. L. B. Rawling, M.B., F.R.C.S., a well-known teacher at "Bart.'s," has collected a good deal of evidence in favour of operations for decompressing the brain in cases of accident or disease where intracranial hæmorrhage has occurred. In giving his views to the Royal Society recently, Mr. Rawling advocated trephining over one of the "silent areas" of the brain, where the sudden relief of pressure cannot harm any particularly important nerve centres. An important point emphasised by this investigator on that occasion was the likely prevention of bad after-symptoms, including headache and epilepsy, such as not infrequently follow severe injuries to the head.

---

Dr. Dudley Corbett, M.A., M.D. Oxon., has lately devised a new method of measuring *x*-ray dosage. This depends on a new form of radiometer, which serves to control the estimations of tint in the Sabouraud's pastilles which are commonly used in *x*-ray work. The new method is said to have given most satisfactory results at St. Thomas's Hospital, where Dr. Corbett is senior assistant in the skin and *x*-ray departments.

---

Teaching in the wards of University College Hospital, Sir John Rose Bradford, M.D., F.R.C.P., F.R.S., has lately been drawing attention to the infrequency with which pericarditis occurs in the typical form described in the text-books; a point of importance considering how firmly the average student pins his faith on the supposed classical signs and symptoms of this condition. Sir John Rose Bradford, besides his active work at University College, holds the posts of post-graduate lecturer at the Seamen's Hospital, Greenwich, Secretary to the Royal Society, and is also a member of the Departmental Committee formed to consider the findings of the Royal Commission on University Education.

---

Dr. E. Poulton and Dr. T. Heaton, who were not long ago appointed medical registrars at Guy's Hospital, are both keen physiological chemists, working under the direction of Dr. Pembrey, the lecturer on physiology there. Dr. Poulton has done a good deal of work on creatin excretion in diabetes, and is now engaged on an investigation into the composition of the alveolar air in that disease; his results so far tend to show how the incidence of diabetic coma is prognosticated by a fall in the percentage carbon dioxide in the depth of the lungs. Dr. Heaton is directing his attention to the general phenomena of metabolism in acute pneumonia.

---

The new representative of the Irish Royal College of Surgeons on the General Medical Council, Sir Lambert H. Ormsby, is a past-president of that institution. The distinguished surgeon qualified L.R.C.P.I. and L.M. in 1869, and has held many surgical posts in Dublin, where he is now honorary surgeon to the Meath Hospital and to the National Children's Hospital. His writings include several contributions on rectal ailments.

---

Mr. Anthony Feiling, M.B., B.C. Cantab., M.R.C.P. London, has been appointed chief assistant for neurology at St. Bartholomew's Hospital, having previously worked in the department of neuro-pathology at Frankfort-on-Main. Mr. Feiling took Honours in the Natural Science Tripos at Cambridge. He has held the post of house physician at St. Bartholomew's and house surgeon at the West London and British Lying-in-Hospitals respectively. He has made a special study of mumps, and has written a very interesting treatise on the subject.

---

At the Cancer Hospital, Fulham, the keen research party has lately been assisted by a visitor from the Continent, Dr. E. P. Minett, whose researches have thrown much light on the life-history and significance of the *micrococcus neoformans.* His results go to show that the famous Doyen was wrong in his well-known theory that this germ is a cause of cancer. A correspondent, who has lately been visiting the chief cancer laboratories in London, points out that this work may not only upset a plausible and popular theory, but put an end to the sale and use of vaccines made for the advertised purpose of destroying the micrococcus neoformans *in situ.*

